# Supplementary material for: Resolving the full spectrum of human genome variation using Linked-Reads
Source: Genome Res. 2019 Apr;29(4):635–45. doi: 10.1101/gr.234443.118 (PMC6442396; doi:10.1101/gr.234443.118)
Supplement: Supplemental Material [file supp_gr.234443.118_Supplemental_longranger-2.2.zip › longranger-2.2.tar.bz2!/lr2.2-public/lib/stan/cmdstan/stan/doc/api/html/globals_defs.html]

Stan: File Members


|  |  |
| --- | --- |
|  | Stan  2.5.0  probability, sampling & optimization |


- Main Page
- Related Pages
- Namespaces
- Classes
- Files

- File List
- File Members

- All
- Functions
- Variables
- Macros


All Classes Namespaces Files Functions Variables Typedefs Enumerations Enumerator Friends Macros Pages

- \_\_STAN\_\_AGRAD\_\_REV\_\_VAR\_STACK\_CPP\_\_
  : var\_stack.cpp
- \_\_STAN\_\_GM\_\_AST\_DEF\_HPP\_\_
  : ast\_def.cpp
- likely
  : likely.hpp
- unlikely
  : likely.hpp


---

[ Stan Home Page ]

*© 2011–2014,
Stan Development Team.*
